# Retraction Note: LncRNA NDRG1 aggravates osteosarcoma progression and regulates the PI3K/AKT pathway by sponging miR-96-5p

**DOI:** 10.1186/s12885-023-11644-1

**Published:** 2023-11-17

**Authors:** Zhen Wang, Yanting Wei, Hao Zhu, Lingfeng Yu, Jie Zhu, Qixiu Han, Ziying Liu, Jianhao Huang, Yan Zhu, Gentao Fan, Qikai Tang, Ji Qian, Xi Chen, Guangxin Zhou

**Affiliations:** 1 Department of Orthopaedics, Jinling Hospital, Nanjing Medical University, Zhongshan Road 305, Nanjing, 210002 China; 2 Department of Epidemiology and Biostatistics, School of Public Health, Nanjing Medical University, Tianyuan East Road 818, Nanjing, 211166 China; 3 Department of Orthopaedics, Affiliated Jianhu Hospital of Nantong University, Yancheng, China; 4 Department of Orthopaedics, Jinling Hospital, Nanjing University, Nanjing, China; 5 Department of Orthopaedics, Jinling Hospital, Southern Medical University, Nanjing, China; 6 Department of Neurosurgery, Jiangsu Province Hospital and Nanjing Medical University First Affiliated Hospital, Nanjing, China; 7 Department of Digestion Medicine, Affiliated Yixing Hospital of Jiangsu University, Yixing, China

**Retraction Note: BMC Cancer (2022) 22:728**

10.1186/s12885-022-09833-5

The Editors have retracted this article. After publication, the authors contacted the journal to request a correction to Fig. 2c, as the same images were used for EdU si-NC and EdU si-Lnc-NDRG1, and provided the raw data for validation. Further checks by the publisher found additional concerns:

Figure 5d MG63 si-NDRG1 + miR-96-5p inhibitor NC and si-NDRG1 + miR-96-5p inhibitor images (and the associated raw data) appear to overlap.

There appear to be several other instances of image overlap in the raw data files.

The Editors therefore no longer have confidence in the presented data.

Xi Chen, Hao Zhu, Guangxin Zhou and Zhen Wang agree to this retraction. None of the other co-authors have responded to any correspondence from the Editors about this retraction.

